# Chronic Traumatic Ulcer: A Case Report

**DOI:** 10.7759/cureus.60774

**Published:** 2024-05-21

**Authors:** Prasanna R Sonar, Aarati Panchbhai, Gunmeek Kaur, Meena Jain, Archana Singh, Teenu Thomas

**Affiliations:** 1 Oral Medicine and Radiology, Sharad Pawar Dental College and Hospital, Datta Meghe Institute of Higher Education and Research (Deemed to be University), Wardha, IND; 2 Oral and Maxillofacial Surgery, Luxmi Bai Dental College and Hospital, Patiala, IND; 3 Public Health Dentistry, Santosh Dental College, Ghaziabad, IND; 4 Pediatric Dentistry, Sardar Patel Post Graduate Institute of Dental and Medical Sciences, Lucknow, IND; 5 Public Health Dentistry, Mahe Institute of Dental Sciences & Hospital, Mahe, IND

**Keywords:** invasive treatment, differential diagnosis, ozonated oil, tongue ulcer, chronic traumatic ulcer

## Abstract

Chronic traumatic ulcers (CTUs) of the oral cavity are frequently brought on by repeated mechanical stress, such as biting or friction from dental appliances, or sharp or broken carious teeth. Although they are frequently disregarded, patients with nonhealing ulcers in the mouth should have CTUs taken into consideration. This report highlights the significance of differential diagnosis and suitable treatment options by discussing a case of a CTU that persisted on the tongue's right lateral border.

## Introduction

Oral ulcers are lesions with a variety of underlying etiologic causes. The tongue, lower lips, and buccal mucosa are the prevalent sites for traumatic ulcers [1]. They could be the consequence of thermal, chemical, or physical trauma. Due to incorrect alignment, sharp edges from decay or fractures, or faulty restorations, carious teeth can alter soft tissue in the mouth [2]. With a wipeable yellowish-white necrotic pseudo membrane, its slightly elevated and reddish edges are coated. Traumatic ulcerations most commonly affect the tongue, lips, and buccal mucosa. Ulcers caused by trauma are more prevalent in men than in women [3]. The frequent damage caused by tongue movement leads to ulcers, which may last for weeks and days, generally heal in 10 days, and usually remain painless for an initial three days [3]. An ulcer with uneven borders, a slightly concave yellowish base, occasionally with induration, and an oval shape are the typical features of chronic traumatic ulcers (CTUs) [4-6].

It can be difficult to diagnose an isolated mouth ulcer. There are several etiologies for oral ulcers that might manifest differently [7]. Both systemic and local factors might result in ulcer formation. Even though an oral ulcer is typically benign, treating oral squamous cell carcinoma (OSCC) has to take precedence in this situation, even if the patient's clinical condition seems encouraging [2]. This report discusses an individual with a tongue ulcer whose clinical characteristics indicate malignancy.

## Case presentation

**Figure 1:**
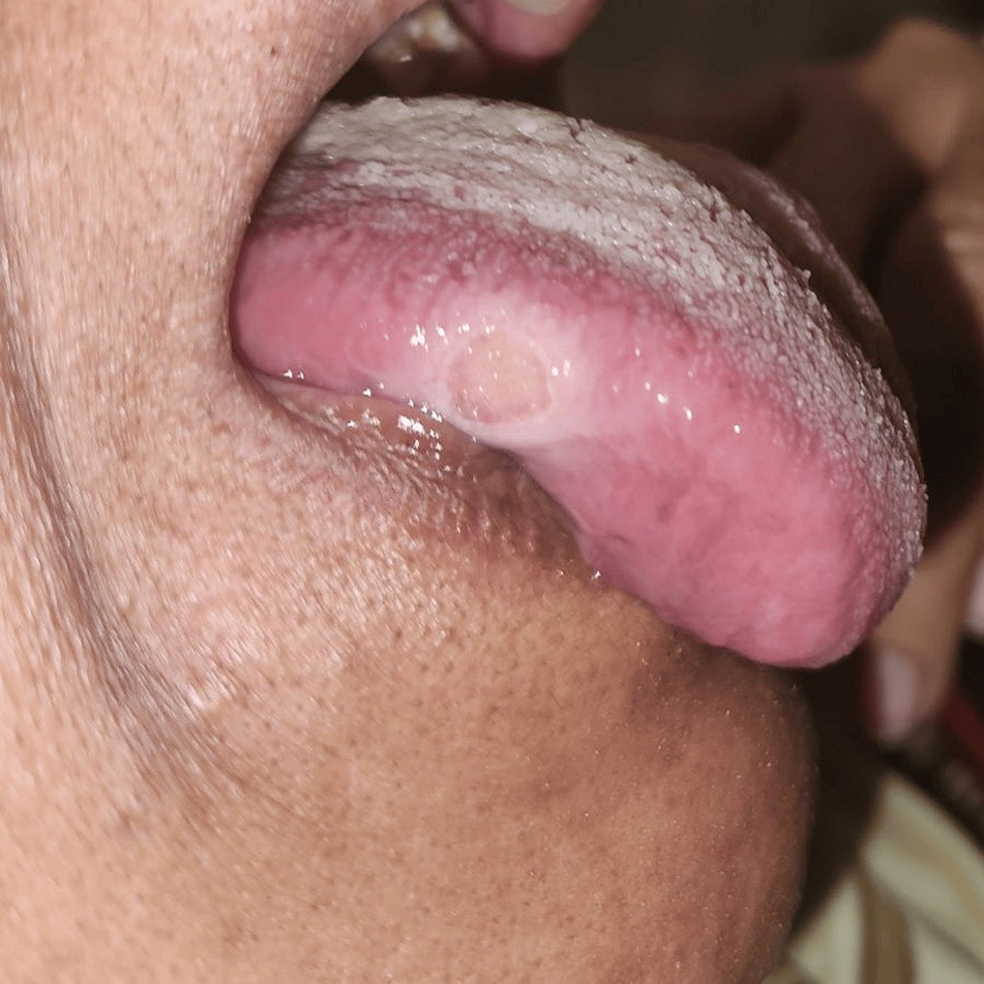
Clinical photograph of the ulcer Image credit: Prasanna Sonar

A 72-year-old woman reported having pain for five months on the right lateral border of her tongue. The persistent pain made it difficult to eat, particularly when consuming spicy or hot foods. The patient had difficulty mastication as well as excruciating discomfort that was exacerbated by food and teeth contact. She didn't smoke or misuse alcohol in the past. Initially little, the ulcer grew to its current size over time. Upon intraoral examination, a single, 1 × 1 cm ulcer was found; aside from its border, the ulcer's hue was similar to the surrounding mucosa. The ulcer had a raised border contrasting with the surrounding tissue's color. As seen in Figure 1, there was no evidence of blood loss or suppuration. It felt stiff to the touch, its base attached to the underlying structures, and its edges indurated and keratotic. Complete blood count and absolute eosinophil count were within the normal range. A provisional diagnosis of the traumatic ulcer was given because there were some sharp teeth cusps in the 45 and 46 regions where the ulcer was found and because they came into contact with the lingual cusp of 45 and 46 in relation to the right lateral border of the tongue. However, given that it manifests clinically as a large ulcer surrounded by induration and hyperkeratosis, we suggested considering OSCC as a differential diagnosis.

**Figure 2:**
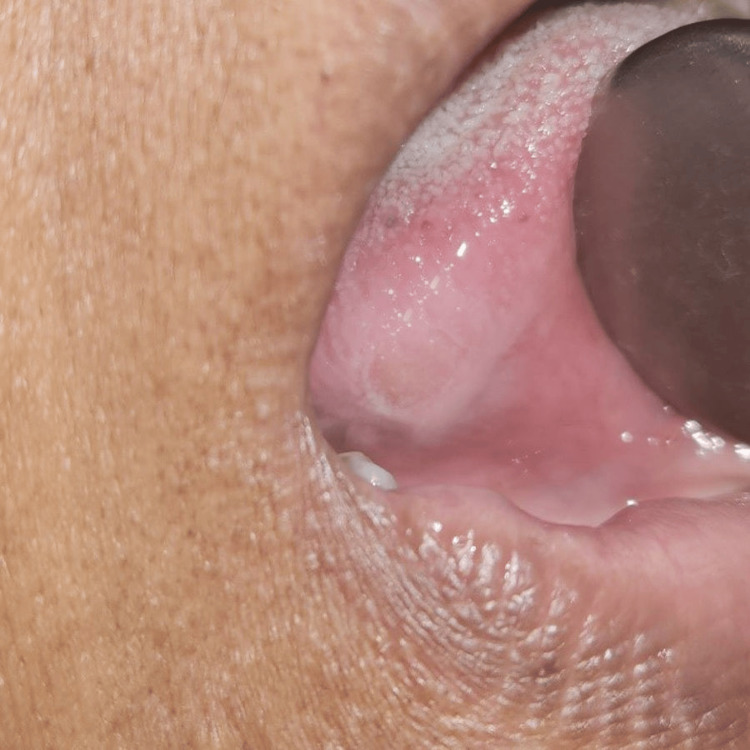
First follow-up visit on the third day Image credit: Prasanna Sonar

**Figure 3:**
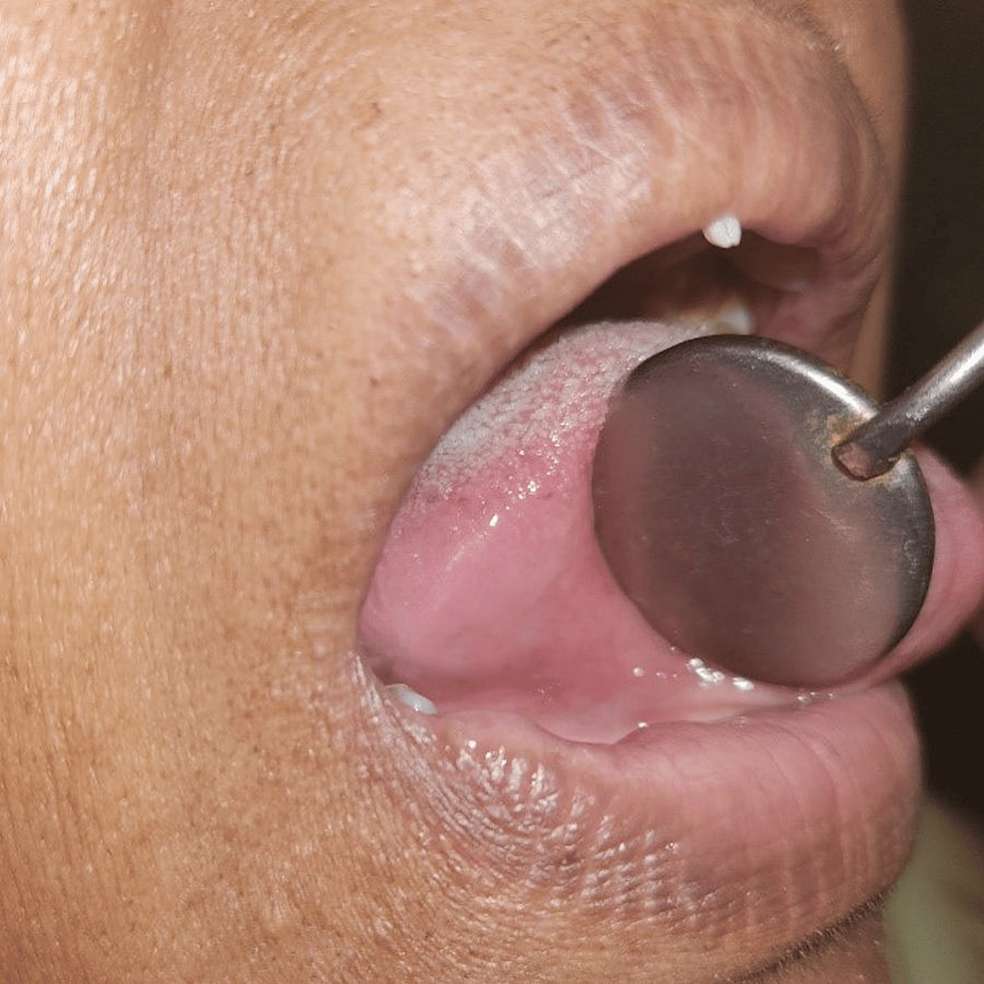
Second follow-up visit on the fifth day Image credit: Prasanna Sonar

We chose to use ozonated oil (ADC Inc. DentozoneIndia) for topical administration because a review of the literature indicated that it improves wound healing [8]. The patient was advised to isolate the ulcer, dry it and apply ozonated oil three to five times a day till the ulcer heals completely. We intended to apply topical ozonated oil after grind the sharp cusp on tooth and monitor the wound till it healed. Following the cuspal grinding of the 45 and 46 teeth, the ulcer vanished five days later, as seen in Figures 2-3.

## Discussion

Ulcer is a break in the skin or mucous membrane with loss of surface tissue, disintegration, and necrosis of the epithelial tissue. The simple definition of an ulcer is the existence of a painful or sore wound. Oral ulcers are lesions that damage the epithelium and the connective tissue underneath. They are characterized by a loss of texture. Since they are ulcerated, they stand out from other lesions and are indicative of a significant histological involvement of the oral mucosa [9]. An ulcer's progression is its most significant feature. An ulcer typically goes through three stages: extension, transition, and healing or repair [10]. Patients seek care for acute ulcers because they hurt and often recover in less than two weeks. An ulcer may develop into a chronic condition if its underlying cause is not resolved. It becomes recurrent, exophytic, and persistent. The underlying cause of an ulcer should be treated; otherwise, it can become chronic, reactive, and exophytic [3]. When an ulcer persists over time and exhibits any induration, it may indicate OSCC [11]. A thorough medical history and clinical evaluation including inspection and palpation are required to identify the source of an oral ulcer. If additional investigations are required, they can be incorporated into the process. Diagnosing traumatic ulcers can be challenging, particularly in cases when there has been no prior trauma. Self-inflicted injuries resulting from parafunctional habits are rare and difficult to diagnose. Under such conditions, the traumatic cause of oral lesions can be disregarded and treated incorrectly [7].

The patient in our instance did not mention any recent traumatic experiences. Nevertheless, we were able to identify the tooth's sharp edge next to the ulcer, which was the actual cause of the ulcer, through a thorough clinical examination. After the trauma's underlying cause was identified and addressed by doing coronoplasty with the associated teeth, the ulcer healed fully. This study emphasizes how crucial it is to identify local causes of mouth ulcers before addressing systemic ones. Traumatic ulcers can have a variety of appearances and might be confusing with other types of oral ulcers, particularly OSCC. In traumatic ulcers, the margin of hyperkeratosis and induration can resemble OSCC [12]. Oral ulcers that may have traumatized origins are not biopsied. Traumatic ulcers should go away if the injury-causing component is removed after two weeks. This period could be prolonged, especially in those with compromised immune systems. Any alarming lesions such as nonhealing ulcers which don't heal for more than four weeks require a histological evaluation [13].

Clinically, the differential diagnosis of CTUs on the tongue is directed toward other isolated chronic mouth ulcers, including mycotic ulcers (deep mycosis), bacterial ulcers (secondary syphilis, tuberculosis), autoimmune ulcers (type: major aphthous stomatitis), and tongue injuries linked to the eruption of primary lower incisors (Riga-Fede disease). Fungal infections, typically caused by *Aspergillus* species or *Candida albicans*, are the cause of mycotic ulcers. These ulcers may have an erythema (redness) surrounding them and appear white or yellowish in hue. They ulcers have a pseudomembranous appearance similar to cottage cheese. Bacterial infections, usually resulting from trauma or tissue injury, are the cause of bacterial ulcers. Bacterial ulcers can have surrounding erythema (redness) and inflammation, and they can present as shallow to deep ulcerations. They can cause pain and have inconsistent borders. There may occasionally be pus or exudate visible. The presentation of autoimmune ulcers might vary based on the particular type of autoimmune disease. They may manifest as painful, deep ulcers with erratic borders (as in pemphigus vulgaris) or as white, lacy patches (as in oral lichen planus) [3,7,10,14]. A traumatic ulcerative granuloma with stromal eosinophilia (TUGSE), which most usually affects the tongue, is another pathological entity to be taken into account in the differential diagnosis of oral ulcers. TUGSE is characterized by a substantial increase in the eosinophil count. It is distinguished by its fast development rate, somewhat indurated boundaries, similarities to OSCC, deep fungal infection, and/or traumatic ulceration. Under the microscope, it can be distinguished from CTU by its intact, well-differentiated epithelium, strong eosinophilia, and a strong inflammatory cell infiltrate [14].

In this case study, the patient's age required prompt therapy to resolve his tongue lesions. While a surgical excision would have undoubtedly been quicker, the ongoing malocclusion would have made stable healing unlikely. In any case, the last approach involves invasive surgery. The patient agreed with our decision to avoid surgery. Over time, the patient's lesion healed completely as a result of steady improvement and results. In this instance, topical ozonated oil treatment and the elimination of the underlying cause made the course of treatment successful. The healing of this tongue CTU indicates that if the irritant cause can be removed, early dysplastic lesions might be reversible [15].

Growth factors, saliva, and secretory immunoglobulin A all promote the spontaneous repair of ulcers [16]. Short courses of topical corticosteroids (two to six days) are particularly recommended for oral mucosal disorders, which typically resolve on their own after the underlying cause is eliminated [17]. The usefulness of hyaluronic acid (HA) in treating mouth ulcers is rarely documented. Due to its role in tissue growth, development, and repair, HA has become more significant in recent years [18]. *Jatropha multifida* herbal ingredients can also be utilized as substitute medications [16].The oral mucosa's ulcers heal more quickly and are less painful when CO_2_ laser therapy is used. Precision targeting, sealing of nerve endings, promoting healing, and a lower risk of infection are all provided by CO_2_ laser therapy, which is minimally invasive [19]. Because of the discomfort, swelling, and bleeding caused by the ulcers, people frequently neglect their dental hygiene, which exacerbates erosive disease and leads to poor plaque control [7].

Oral ulcers should not be biopsied as having traumatic etiology. After the injury's contributing component is eliminated, traumatic ulcers should heal in two weeks. This time frame may extend, especially in those with compromised immune systems. A histological analysis is required for every concerning lesion or nonhealing ulcer [20]. It is preferable to take an incisional biopsy for tiny ulcers (5 mm in diameter). A portion of the ulcer and the perilesional tissue, including the unaffected surrounding epithelium, must be included in the specimen. Typically, the ulcer's center by itself lacks diagnostic characteristics. It is recommended to use punch or scalpel biopsies instead of laser or electrical scalpels [3]. The histological architecture of the tissue is typically preserved more effectively by punch and scalpel biopsies than by laser or electrosurgical techniques. Thermal injury to the tissue brought on by laser and electrosurgical equipment may produce artifacts like coagulation, charring, or heat-induced necrosis. These artifacts might mask histological characteristics, making proper tissue interpretation challenging. Preserving the architecture of tissues is essential for precise histological diagnosis. This is especially crucial when considering infectious, neoplastic, or inflammatory etiologies since heat injury can obscure cellular information that is essential for a diagnosis.

In addition to prioritizing advanced studies, a holistic approach, and early diagnosis of oral lesions, all patients should focus on controlling oral hygiene and eliminating the cause of irritation to the oral mucosa. Dentists and oral medicine specialists have the ability to greatly enhance and maybe improve a patient's life. It is crucial to know early identification of mucosal lesions and oral health care.

## Conclusions

This report of a CTU on the tongue concludes by emphasizing the value of a comprehensive clinical examination in distinguishing between common benign lesions and more serious illnesses. The prompt presentation and comprehensive medical history provided by the patient were essential in determining the traumatic etiology, which was linked to unintentional biting and potentially exacerbated by dental etiology such as dental appliances, restorations, or prostheses. Treatment with conservative measures, such as avoiding the trauma that caused it, maintaining good oral hygiene, and using protective agents topically, resolved the symptoms in two weeks, proving that noninvasive approaches to treating these lesions are effective. This case further emphasizes how important it is for medical professionals to take patient-specific characteristics into account while diagnosing and treating oral lesions, such as sharp tooth cusps. It helps to warn people of the risk of incorrect diagnosis and the ensuing inappropriate therapy that can result from failing to carefully evaluate and take into account each aspect of the patient's clinical presentation and medical history. Future advice should focus on educating patients on how to take care of their teeth and gums and stress the value of periodic dental checkups to avoid situations like this one.
